# Dietary Arsenic Exposure in Bangladesh

**DOI:** 10.1289/ehp.9462

**Published:** 2007-02-20

**Authors:** Molly L. Kile, E. Andres Houseman, Carrie V. Breton, Thomas Smith, Quazi Quamruzzaman, Mahmuder Rahman, Golam Mahiuddin, David C. Christiani

**Affiliations:** 1 Harvard School of Public Health, Boston, Massachusetts, USA; 2 Dhaka Community Hospital, Dhaka, Bangladesh

**Keywords:** arsenic, Bangladesh, dose, duplicate diet, food, intake, water

## Abstract

**Background:**

Millions of people in Bangladesh are at risk of chronic arsenic toxicity from drinking contaminated groundwater, but little is known about diet as an additional source of As exposure.

**Methods:**

We employed a duplicate diet survey to quantify daily As intake in 47 women residing in Pabna, Bangladesh. All samples were analyzed for total As, and a subset of 35 samples were measured for inorganic arsenic (iAs) using inductively coupled plasma mass spectrometry equipped with a dynamic reaction cell.

**Results:**

Median daily total As intake was 48 μg As/day [interquartile range (IQR), 33–67) from food and 4 μg As/day (IQR, 2–152) from drinking water. On average, iAs comprised 82% of the total As detected in dietary samples. After adjusting for the estimated inorganic fraction, 34% [95% confidence interval (CI), 21–49%] of all participants exceeded the World Health Organization’s provisional tolerable daily intake (PTDI) of 2.1 μg As/kg-day. Two of the 33 women who used a well with < 50 μg As/L exceeded this recommendation.

**Conclusions:**

When drinking water concentrations exceeded the Bangladesh drinking water standard of 50 μg As/L, ingested water was the dominant source of exposure. However, as drinking water As concentrations decrease, the relative contribution of dietary As sources becomes more important to ingested dose. The combined intake from both diet and drinking water can cause some individuals to exceed the PTDI in spite of using a tube well that contains < 50 μg As/L.

Use of groundwater has reduced the morbidity and mortality from waterborne disease in Bangladesh and helped the country achieve self-sufficiency in cereal production through dry-season irrigation (Ahmad 2001; Gill et al. 2003). However, the shallow groundwater aquifer of this region is highly contaminated with naturally occurring arsenic from dissolved minerals and ores. In a national survey conducted by the British Geological Survey and the Department of Public Health Engineering, Bangladesh (BGS and DPHE 2001), 27% of the shallow tube wells exceeded the Bangladesh drinking water standard of 50 μg/L, exposing an estimated 33 million people to potentially dangerous levels of As in their drinking water. Chronic exposure to As increases the risk for As-induced diseases such as noncancerous skin lesions, bronchitis, hepatomegaly, neuropathy, peripheral vascular diseases (e.g., gangrene), cardiovascular disease, skin cancer, lung cancer, and bladder cancer (Chen and Ahsan 2004; Chowdhury et al. 2000; Mazumder 2003; McLellan 2002; Smith et al. 1998).

Although there is no question that consumption of As-contaminated drinking water is the most important route of exposure in Bangladesh, little research has focused on food as an additional source of exposure in spite of evidence that rice, a dietary staple, can accumulate As when grown in contaminated environments. Studies have shown that irrigation with As-contaminated water can lead to elevated As concentrations in rice-paddy soil, as well as in the rice root, stalk, and grain (Duxbury et al. 2003; Meharg and Rahman 2003; Norra et al. 2005). Market basket studies that analyze individual food items also found that As concentrations in commonly consumed vegetables are directly correlated with the As concentration in irrigation water (Alam et al. 2003). Furthermore, cooking with As-contaminated water can be an additional source of exposure because rice absorbs twice its weight in water when cooked (Bae et al. 2002).

To more fully understand the relative contribution of food and drinking water to ingested As dose, we conducted a duplicate diet study in Pabna district, located north of Dhaka in central Bangladesh. We targeted female heads of households because they are responsible for all food preparation; they also represent a potentially susceptible sub-population because there is mounting evidence that As is transmitted to the fetus (Jin et al. 2006) and can influence child neurodevelopment (Wasserman et al. 2004; Wright et al. 2006).

## Methods

### Participant selection

Forty-seven women who had previously taken part in a longitudinal study investigating As exposure and biomarker response, and who identified themselves as the primary food preparer in the family were invited to participate in this study (Kile et al. 2005). All women agreed to participate, and informed consent was obtained. Two sampling periods were scheduled for 3 consecutive days in winter (January–March 2004) and for 3 consecutive days in summer (June–August 2004). Participants received compensation (US$9) after each sampling period. The institutional review boards at Harvard School of Public Health and Dhaka Community Hospital approved the protocol for this study.

### Food samples

Participants were instructed to save duplicate portions from each meal in separate polypropylene resealable bags. Researchers visited each participant after the midday and evening meals to collect samples, which were kept refrigerated until processing. Each evening, individual portions were weighed in order to determine dietary intake rates. The portions from each participant were then homogenized into a 24-hr composite sample (*n* = 282) using a blender. Homogenized samples were aliquotted into polyethylene tubes and frozen at –4°C and shipped on dry ice.

Field blanks, composed of 50 mL Milli-Q 18.2 Ω analytical grade water (Millipore Corporation, Billerica, MA, USA), were collected after every eighth sample. For the field blanks, the water underwent the same homogenization process as the food samples and was preserved with reagent grade nitric acid (Merck, Darmstadt, Germany) to a pH of < 2. Composite samples were subjected to microwave acid digestion with nitric acid and analyzed for total As using dynamic reaction cell inductively coupled plasma-mass spectrometry (ICP-DRC-MS) with oxygen as the cell gas (Model 6100 DRC; PerkinElmer, Norwalk, CT, USA). This analytical method detects As oxide species (^75^As^16^O^+^) at mass 91 and avoids argon chloride interference (Bollinger and Schleisman 1999). Indium was added as an internal standard upstream of the nebulizer. The average limit of detection (LOD) was 0.07 μg As/L. The average field blank (± SD) contained 0.03 ± 0.07 μg As/L, with only six field blanks exceeding the average LOD. Each sample was analyzed five times, and the reported total As value was corrected for any As detected in the method blank and field blank. Four samples had corrected total As concentrations below the averaged method LOD of 0.07 μg/L and were assigned half the LOD.

Samples were digested in 14 batches, with each batch containing a method blank, certified rice flour [standard reference material (SRM) 1568A; National Institute of Standards and Technology (NIST), Gaithersburg, MD, USA] and certified dogfish liver sample (DOLT-2; National Research Council, Ottawa, Ontario, Canada). The average percent recovery (± SD) was 102.2 ± 7.9% for SRM 1568A and 93.0± 7.1% for DOLT-2. We used SRM 1643e (Trace Elements in Water; NIST) to validate instrument performance. The average percent recovery for SRM 1643e was 101.7 ± 5.8%. Additionally, 10% of the samples were randomly chosen for replicate analysis and were analyzed on separate days in the laboratory. The average percent difference in As concentrations detected in replicate samples was 4.0%.

Thirty-five samples representing a range of drinking water exposures were analyzed by Brooks Rand (Seattle, WA, USA) for total As and inorganic arsenic (iAs). This served as an interlaboratory validation for total As and also allowed for the estimation of the iAs fraction in composite dietary samples. Three samples partially thawed during shipping; however, it is unlikely that this brief warming influenced As speciation because it lasted < 24 hr. Brooks Rand extracted the 35 samples for total As and iAs following modifications of U.S. Environmental Protection Agency (EPA) methods 1638 (U.S. EPA 1995b) and 1632 (U.S. EPA 1995a), respectively. For total As, samples were closed-vessel oven-bomb digested with concentrated nitric acid and analyzed by ICP-DRC-MS. For total iAs, sample aliquots were extracted with hydrochloric acid and the pH adjusted to 1.5 before analysis. The comparison between laboratories for total As was good, with an interlaboratory percent difference of 14.6 ± 19.1%.

The quality control at Brooks Rand included method blanks, certified dogfish muscle tissue (DORM-2; National Research Council, Canada), dogfish liver tissue (DOLT-3; National Research Council, Canada), and certified lobster hepatopancreas (TORT-1; National Research Council, Canada), spiked reference material, and matrix spikes at concentrations > 10 times the native sample concentration. The percent recovery for DORM-2, DOLT-3, and TORT-1 was 93%, 87%, and 103% respectively. The average percent recovery (± SD) for total As from two spiked certified material samples, run in triplicate, was 92.6 ± 7.3% and the average recovery for iAs was 96.8 ± 11.9% and 91.5 ± 4.5%. Water (SRM 1640 and SRM 1643e; NIST) was used to validate instrument performance; the percent recoveries for SRM 1640 and SRM 1643e were 112% and 102%, respectively. All quality assurance met the laboratory’s acceptance criteria. The method LODs for total As and iAs were 0.013 and 0.003 μg/g, respectively. All samples were above the LODs.

### Drinking water

All women reported having their own tube well and using it for all water for drinking and cooking purposes. Participants were provided with two 4-L polyethylene containers and instructed to place an identical quantity of water in the containers immediately after drinking a glass of water in order to determine drinking water intake rates. However, the concentration of As in each participant’s tube well was estimated using water samples collected as part of a larger, longitudinal biomarker study in which water samples were collected for 3 consecutive days every 3 months (Kile et al. 2005). Data from 2004, representing 12 samples per participant and overlapping the time frame of this duplicate diet study, were used to establish an average annual drinking water As concentration for each participant. Total As was measured by Environmental Laboratory Services (North Syracuse, NY, USA) using ICP-MS following U.S. EPA method 200.8 (U.S. EPA 1994).

Quality control criteria included analyzing Plasma CAL#1 Multi-Element QC Standard (SCP Science, Baie D’Urfè, Quebec, Canada). The average percent recovery (± SD) was 96.7 ± 3.4%. Additionally, 10% of the samples were randomly chosen for replicate analysis, with the average percent difference in As concentrations of 0.2%. Twenty-one households had drinking water As concentrations below the 1-μg/L LOD and were assigned half the LOD.

### Statistical analysis

We recorded the weight of each duplicate meal portion (grams wet weight) and the volume of drinking water (milliliters) collected in a 24-hr period as the daily dietary intake rate and drinking water intake rate, respectively. Descriptive statistics were calculated, including mean ± SD. We calculated daily total As intake from food (micrograms per day) for each participant using the total wet weight of food consumed each day multiplied by the total As concentration measured in the corresponding 24-hr composite sample (micrograms per gram wet weight). We multiplied the annual average total As concentration in each participant’s tube well by the total volume of water consumed each day to determine daily total As intake from water (micrograms per day) from drinking water. The daily total As intake was the sum of daily total As intake from food and drinking water. Dividing each participant’s daily As intake by their body weight determined the daily total As dose (micrograms per kilogram per day). Medians and interquartile range (IQR; 75th percentile – 25th percentile) were reported for all exposure outcomes.

Because drinking water As concentrations were positively skewed, they were subsequently transformed to their common logarithms. We used generalized estimating equations (GEE) employing an exchangeable working correlation structure to evaluate seasonal and daily differences in As concentrations in food composite samples and dietary and drinking water intake rates. We used two regression techniques, GEE and median regression, to examine the relationship between As-contaminated drinking water and dietary As intake, which approximates the effect of preparing and cooking food with contaminated water. The median regression technique, which is robust to outliers, estimated standard errors using the resampling method while taking into account repeated measures (Parzen et al. 1994).

We estimated the iAs fraction using diet samples with both total As and iAs measured by the same laboratory. The average iAs fraction was then multiplied by the average total As dose from food and summed with the daily As dose from drinking water in order to estimate the amount of iAs ingested. We then compared these values with the World Health Organization’s (WHO) provisional tolerable daily intake recommendations (WHO 1985). We report the proportion of participants whose average daily iAs dose exceeded the WHO’s PTDI of 2.1 μg/kg-day along with exact confidence intervals (CIs). All statistics were computed using SAS for Windows, version 9.1(SAS Institute Inc., Cary, NC, USA) except the median regression analysis which was conducted in R, version 2.0.1 (R Foundation for Statistical Computing 2006).

## Results

The participant’s physical and demographic characteristics are presented in Table 1. The median drinking water concentration for the 47 tube wells sampled was 1.6 μg/L (range, < 1–450 μg/L). Overall, 60% were below the WHO’s 10-μg/L drinking water standard (WHO 1985), and 70% were below the Bangladesh drinking water standard of 50 μg/L (BGS and DPHE 2001). On average, participants consumed 1,636 g food (wet weight) and 2,676 mL water per day. Participants consumed significantly more food in winter (1,700 ± 338 g wet weight) than in summer (1,571 ± 324 g wet weight), but no seasonal difference was detected in the concentration of As in the composite food samples. The number of servings collected did not vary significantly over the course of the study. Also, we did not find a significant difference in the amount of food collected within each season. No seasonal or daily difference was observed in the drinking water intake rate.

The frequency of each food type collected in the duplicate diet study is shown in Table 2. Vegetables and rice were the most commonly consumed food items. Rice, the dietary staple, was present in 91% of all collected meals, with 405 g (wet weight) consumed in an average serving. Vegetables were present in 94% of all meals collected, with an average serving size of 72 g wet weight. Freshwater fish was the most commonly consumed protein. Pabna is far enough inland that seafood is not readily available in the local markets, and no participants reported eating either seafood or shrimp during this study period. Furthermore, all participants reported purchasing their food at local markets. These items would most likely be produced domestically, if not locally. However, this data was not collected.

**Figure 1:**
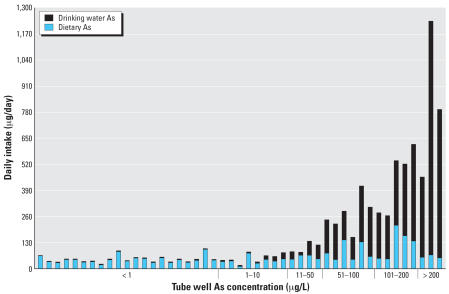
Distribution of average daily As intake (μg/day) from both drinking water and dietary sources for all 47 participants sorted by tube well As concentration.

The distribution of total dietary As intake and dose were heavily skewed, driven by the overwhelming contribution from contaminated drinking water for the upper 25th percentile of the population (Figure 1). When drinking water As concentrations decreased, the relative contribution of As from dietary sources increased. Background dietary total As intake for the population, calculated using the dietary exposures for the participants with no detectable As in their drinking water, was 46 μg/day or 0.91 μg/kg-day. For all participants, the combined median daily total As intake from both food and drinking water was 68 μg/day (IQR, 191 μg/day). The median daily total As intake from food only was 48 μg/day (IQR, 34 μg/day) and drinking water only was 4 μg/day (IQR, 150 μg/day).

A subset of 35 samples (12% of the total sample collected) analyzed for both total As and iAs were used to estimate the iAs fraction in the 24-hr dietary composite samples. The average inorganic fraction (± SD) in dietary samples was 82.1 ± 13.9%. Linear regression showed that iAs explained 90% of the variability in total As measurements. To estimate the daily iAs dose, all dietary doses were adjusted by the inorganic fraction before being added to the drinking water doses because it is assumed that all As present in drinking water is in the inorganic form. These values were compared to the WHO’s iAs PTDI of 2.1 μg/kg-day (WHO 1985). Overall, 34% (95% CI, 21–49%) of all participants had an average daily dose that exceeded this recommended limit. Of the four women who used tube wells containing 10–50 μg As/L, two exceeded the PTDI. For women who used a tube well containing < 10 μg As/L, diet was the only substantial source of ingested As.

**Figure 2:**
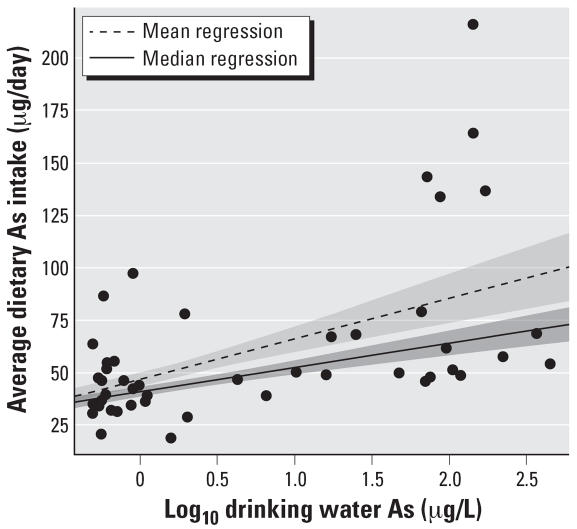
Average dietary As intake (μg/day) plotted against the logarithm of drinking water As (μg/L). The mean and median regressions were obtained from the model *Y* = (α + β) × (log_10_ *X* + ɛ), where either the mean or median of ɛ is zero. For the median regression line, α = 40.5 and β = 11.6; for the mean regression line, α = 46.0 and β = 19.5. Shaded areas represent 1 SE around each regression line.

Using both GEE and median regression models, we found a significant association between the concentration of As in a given household’s drinking water and the total As concentration measured in their food (Figure 2). This likely reflects the effect of cooking and preparing food with As-contaminated water. The median regression model provided the best fit to the average dietary total As intake, as indicated by the smaller SE. This model estimated that dietary total As exposure increased by 0.5 μg/day (95% CI, 0.2–0.7 μg/day) for every 10% increase in drinking water As concentration.

It is interesting to note that only one participant was diagnosed with As-induced skin lesions (melanosis, leukomelanosis, and hyperkeratosis of the palms and soles). This 38-year-old woman reported using the same tube well—one with an average As concentration of 360 μg/L—for the past 12 years. She had the highest observed average daily total As intake (1231.3 μg/day) and subsequent average daily total As dose (25.7 μg/kg-day). However, another participant with no visible As-induced skin lesions had a higher well concentration. This reinforced the notion that interindividual differences in ingestion rates and duration of exposure are an important contributing factor in exposure assessments.

## Discussion

In Bangladesh, groundwater provides 95% of the drinking water and approximately 71% of the agricultural irrigation water (Fazal et al. 2001). The shallow aquifer beneath Bangladesh is contaminated with naturally occurring As, and chronic As exposure is widespread throughout the country. Arsenic exposure from drinking contaminated water has received the most attention, primarily because of the high As concentrations detected but also because of the circumstances that generated the current As crisis. In the 1970s, tube wells were installed to switch the population from microbially contaminated surface water to ground-water to decrease the morbidity and mortality from waterborne disease. It was not until 20 years later that the public became aware that these relatively shallow tube wells could be contaminated with As, thus introducing a new health burden on the community.

We observed a median daily total As dose of 1.3 μg/kg-day, whereas the median daily total As dose from drinking water and diet was 0.08 μg/kg-day and 1.0 μg/kg-day, respectively. These exposure estimates reflect the As contamination in our study area and the relative distribution of As-contaminated water. National groundwater surveys show that As concentrations in approximately 27% of tube wells exceed the Bangladesh drinking water standard of 50 μg/L and 51% exceed the WHO drinking water recommendation of 10 μg/L (BGS and DPHE, 2001). We observed a similar distribution in the present study, with 30% of the tube wells containing > 50 μg/L and 40% containing > 10 μg/L. Our exposure assessment suggested that when tube well concentrations were > 50 μgL, water was the dominant route of exposure. However, if the observed distribution of contaminated tube wells is representative, then drinking As-contaminated water will be the dominant route of exposure for only one-third of the population. Dietary sources of As, on the other hand, will be the most important route of exposure for the remaining two-thirds of the population. Therefore, it is important to further understand the health risks associated with this route of exposure.

The average daily total As intake calculated in the present study was 174 μg/day, which is considerably lower than the 515 μg/day estimated in an earlier study for an adult Bangladeshi (Watanabe et al. 2004). This discrepancy could be due to regional differences in As contamination or from methodologic differences between the two study designs, because the Watanabe study employed a market basket technique to estimated food-derived exposure and we used a duplicate diet methodology that analyzed As in cooked, ready-to-eat food. Duplicate diet studies are considered to be more accurate at estimating personal exposures because they account for the individual’s water source, the type and quantity of food items consumed, and the agricultural conditions under which the food is cultivated (WHO 1985). It is important to note that the estimates derived from duplicate diet studies depend on the dietary habits of the participants and may not be generalizable to other populations. Because we collected dietary data from women only, the results may not be generalizable to men because gender influences the intrafamilial distribution of food in Bangladeshi households, with men eating on average, 40% more cereals, 26% more tubers, 29% more pulses, and 57% more vegetables than women (Hassan and Ahmad 1982). Thus, it is possible that adult males may have higher As exposures than women.

In the present analysis, 34% (95% CI, 21–49%) of the participants ingested iAs concentrations in excess of the WHO’s recommended daily allowance of 2.1 μg/kg-day (WHO 1985). If drinking water contained > 50 μg As/L, water was the dominant route of exposure. However, the combined intake from food and drinking water was sufficiently high that two women who used a well containing 10–50 μg/L exceeded this recommended daily allowance. This provides evidence that the current Bangladesh drinking water standard of 50 μg/L might not be protective of public health when all routes of exposure are considered. The sources of As in the diet are likely to be from rice and vegetables cultivated in As-contaminated environments because these are the two types of food items most commonly consumed. However, we also observed that food preparation modestly contributed to dietary As intake, which has been observed in experimental settings (Bae et al. 2002). However, it is important to recognize that the present study is small, and further studies will be required before determining the source of dietary As and whether the exposure estimates computed for this population are generalizable to other regions in Bangladesh.

It is also important to recognize that the fraction of iAs in food items varies widely (Schoof et al. 1999). We estimated that the average iAs concentration comprised 82% of the total As detected in a subset of the dietary samples. This is similar to values reported by Smith et al. (2006) who reported that iAs made up 87% of the total As measured in rice and 96% of the total As measured in vegetables commonly consumed in Bangladesh. Our estimated inorganic fraction is slightly lower, but we computed the inorganic fraction in homogenized 24-hr dietary samples rather than individual food items. Also, the absorbed dose that influences toxicity depends on the solubility of the iAs during gastrointestinal digestion, which is poorly understood and varies with food type.

Biomarker studies provide evidence that dietary sources contribute to internal dose. Studies that have looked at both urinary and toenail As concentrations found that the relationship between these biomarkers and drinking water As concentrations are nonlinear at low drinking water As concentrations but become linear as drinking water As concentrations increase (Karagas et al. 2000; Kile et al. 2005; Watanabe 2001). It is therefore likely that the added exposure from dietary sources explains the observed nonlinearity in these relationships.

Bangladesh is developing rapidly and has become dependent upon groundwater as a source of drinking and irrigation water. While providing safe drinking water to exposed individuals must remain a public health priority, it is also important that irrigation policies are reviewed, because this analysis clearly demonstrates an elevated background exposure from dietary sources. In accordance with the recently adopted national policy for As mitigation, which acknowledges that As in groundwater used for irrigation may also have an effect on the food chain, preference should be given to surface water for irrigation where appropriate (Bangladesh Government 2004). Furthermore, it is important to continue to monitor the food chain because continued use of As-contaminated irrigation water is likely to increase the probability and magnitude of dietary As intake.

## Figures and Tables

**Table 1:** Physical and demographic characteristics of the 47 female participants.

Characteristic	Percent of population	Mean ± SD	Range
Age (years)		36.6 ± 8.6	20–65
Body mass index		22.5 ± 3.5	15.1–30.3
Years using tube well		31.9 ± 7.8	11–54
Years using current tube well		8.7 ± 6.1	1–20
Marital status			
Married	94		
Widowed	6		
Occupation			
Homemaker	96		
Factory worker	2		
Office worker	2		

**Table 2:** Frequency of food types collected in the duplicate diet study.

	Winter [No. (%)]	Summer [No. (%)]	Total [No. (%)]
Grains			
Rice	393 (35.0)	390 (33.7)	783 (34.3)
Bread	32 (2.9)	43 (3.7)	75 (3.3)
Proteins			
Fish (all freshwater)	139 (12.4)	95 (8.2)	234 (10.3)
Meat (poultry, beef, goat)	27 (2.4)	21 (1.8)	48 (2.1)
Egg	21 (1.9)	22 (1.9)	43 (1.9)
Fruits and vegetables			
Vegetables	436 (38.8)	374 (32.4)	810 (35.5)
Fruit	1 (0.1)	80 (6.9)	81 (3.6)
Pulses/legumes	65 (5.8)	94 (8.1)	159 (7.0)
Others			
Condiments (sugar, salt)	3 (0.3)	11 (1.0)	14 (0.6)
Fried snacks	2 (0.2)	4 (0.4)	6 (0.3)
Butter	0 (0)	1 (0.1)	1 (0.0)
Dessert (sweet noodles)	2 (0.2)	7 (0.6)	9 (0.4)
Dairy			
Milk^a^	3 (0.3)	14 (1.2)	17 (0.8)

A total of 432 meals were collected for each season, with 864 meals collected in total from 47 participants.

a Was not included in the 24-hr composite or analyzed for As.
